# Osteopetrosis, Hypophosphatemia, and Phosphaturia in a Young Man: A Case Presentation and Differential Diagnosis

**DOI:** 10.1155/2012/238364

**Published:** 2012-02-09

**Authors:** Zahi Mitri, Vin Tangpricha

**Affiliations:** ^1^Department of Medicine, Emory University School of Medicine, 101 Woodruff Circle NE, Atlanta, GA 30322, USA; ^2^Division of Endocrinology, Metabolism and Lipids, Department of Medicine, School of Medicine and Atlanta VA Medical Center Emory University, Decatur, GA 30300, USA

## Abstract

We report the case of a 30-year-old African-American male with osteopetrosis and hypophosphatemia, presenting with diffuse myalgias. Laboratory evaluation performed revealed a low serum phosphorus level with urinary phosphate wasting, low calcium, and 25-hydroxyvitamin D concentrations, as well as elevated alkaline phosphatase. Skull and pelvic radiographs revealed high bone density consistent with high bone mass found on bone mineral density reports. PHEX gene mutation analysis was negative. Patient was started on calcium and phosphorus replacement, and he clinically improved. This paper will review the different subtypes of osteopetrosis, and the evaluation of hypophosphatemia.

## 1. Introduction

 This manuscript will present the case of a 30-year-old African American male who presented to medical attention with diffuse myalgias and bony pain. The patient was first treated symptomatically for his hip pain which became progressively worse and was associated with hearing loss and visual disturbances. He was referred for further evaluation, when he was found to have a dual diagnosis of osteopetrosis and hypophosphatemia. This article will mainly focus on the diagnostic evaluation and differential for each condition, as well as a potential unifying diagnosis.

## 2. Presentation of Case

A 30-year-old previously healthy gentleman initially presented approximately two years ago prior to our evaluation with right hip pain occurring during military training. He had no history of trauma occurring prior to this event. He was diagnosed with muscle strain and instructed to take pain medications as needed. Two weeks later, he restarted his training, and shortly afterwards developed bilateral hip pain. He also complained of headaches, mild bilateral hearing loss, as well as blurring of vision for which he started wearing glasses. His physical exam was remarkable only for mild frontal bossing and diffuse bony aches. The patient's height was 150 cm, which falls below the fifth percentile for his age group. Initial labs revealed an elevated parathyroid hormone (PTH) (86 pg/mL (15–65 pg/mL)), low serum calcium at 8.7 mg/dL (9–10.5 mg/dL), ionized calcium was 1.2 mmol/L (1.1–1.4 mmol/L), and nonfasting phosphorous was 2.0 mg/dL (2.4–4.5 mg/dL). Albumin level was mildly elevated at 5.02 g/dL (3.0–5.0 g/dL), total protein was 7.02 g/dL (6.4–8.2 g/dL). He also had mild anemia with a hemoglobin concentration of 20.3 g/dL (13.7–17.5 g/dL). Iron studies, serum protein electrophoresis, and hemoglobin electrophoresis were normal. His pelvic radiographs demonstrated increased bone density. A bone density by dual X-ray absorptiometry scan demonstrated a vertebral spine (L1–L4) T score of +7.4 and total hip *T*-score of +4.2. A vertebral fracture assessment demonstrated a moderate compression of T11. The former films did not reveal any focal zone of decreased uptake suggestive of Brown's tumor. A pituitary MRI and parathyroid scan did not reveal a pituitary adenoma or parathyroid adenoma.

His family history is significant for a younger brother with diffuse bone pain and headaches as well low serum phosphorus. His brother has not had any further workup. He reports one cousin and nephew with bowing of legs and short stature and a second cousin who suffers from severe musculoskeletal pain and short stature. No family members have been evaluated for causes of their short stature or musculoskeletal pain. No women are affected in the family, and all affected individuals are from the maternal side.

He was initially prescribed calcium replacement and pain medication. He was also advised to quit the military. The patient did not improve on this treatment and sought a second opinion from a pediatric endocrinologist. The initial tests were repeated, including the bone mineral density, which confirmed the diagnosis of high bone mineral density. Additional testing revealed low serum phosphorous and low 24-hour urine phosphorus. His tubular maximal phosphorous reabsorption per glomerular filtration rate (TMP/GFR) was 1.1 mg/dL (2.6–4.4 mg/dL). He was started on calcitriol and potassium phosphate/sodium phosphate supplements.

**Figure 1:**
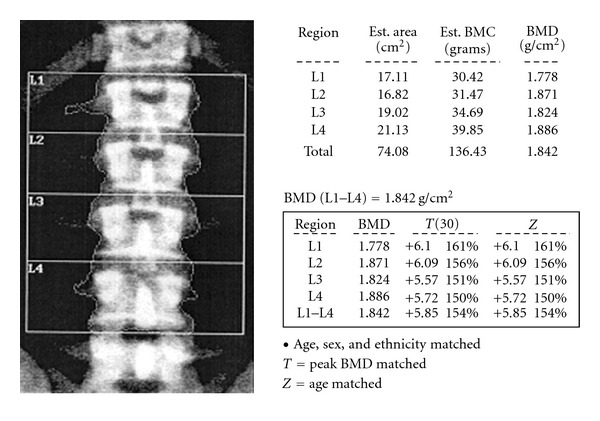
Bone mineral density (BMD) of the lumbar spine. Our patient presented with elevated bone mineral density at the lumbar spine (1.842 g/cm^2^). This value is 5.85 standard deviations above the mean for age- and sex-matched controls (*Z*-score); 5.85 standard deviations above the mean level of peak bone mass (*T*-score).

**Figure 2:**
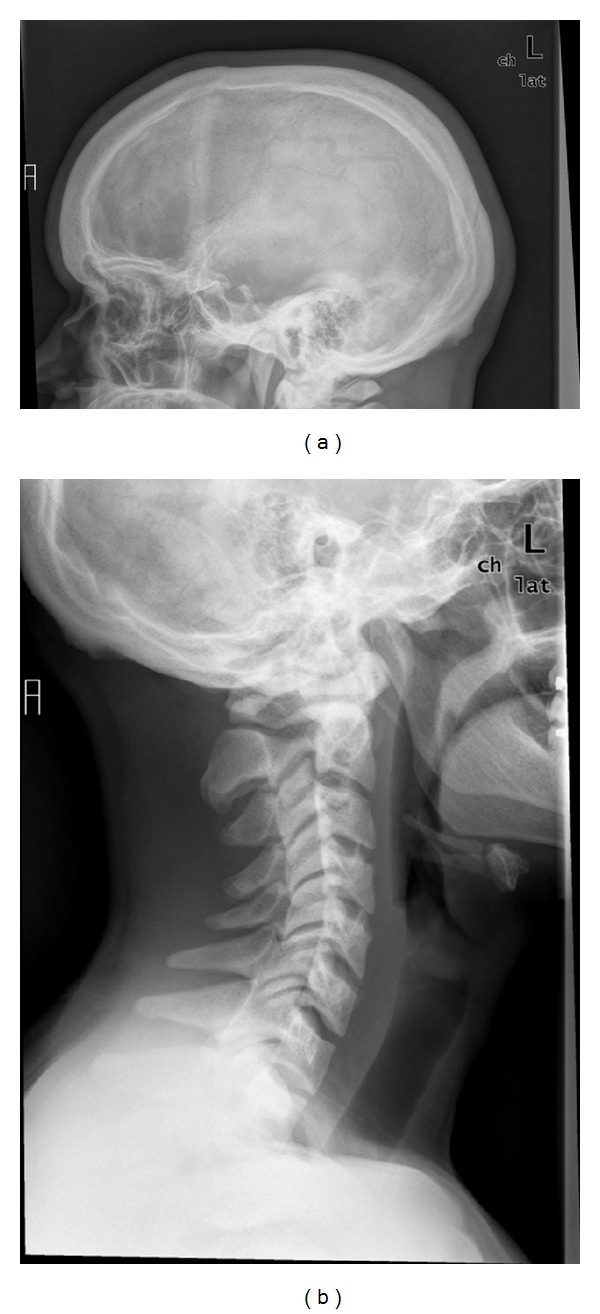
Skull films. Patient's skull films are shown. They are significant for a thickened calvarium and prominent thickening of the skull at the base with a normal and symmetric density. No narrowing of the intervertebral spaces and no fractures are seen.

The patient was referred to our institution approximately two years after initial presentation. A repeat bone density measurement by DEXA revealed high bone density in the total hip (+2.31 *T*-score) and in the vertebral spine (L1–L4) (+5.85 *T*-score) (Figure 1). His skull radiographs were remarkable for a thick calvarium with normal and symmetric density (Figure 2). Skeletal survey and lumbar spine exam were within normal limits. The patient had normal serum calcium (9.7 mg/dL), low fasting serum phosphorous (1.8 mg/dL) (2.4–4.5 mg/dL) and a 24-hour urine collection that demonstrated phosphate wasting (2666.38 mg/24 hours) (800–2000 mg/24 hours). His 25-hydroxyvitamin D was 17 ng/mL (30–100 ng/mL). His PTH concentration was within normal limits at 46 pg/mL (15–65 pg/mL), and alkaline phosphatase level was elevated at 139 IU/L (30–115 IU/L). Hypophosphatemia was further investigated with DNA sequencing, looking for a potential PHEX gene mutation. Twenty-two segments of the PHEX sequence were found to be negative for a significant PHEX mutation. 

 He was started on calcitriol 0.25 mcg twice daily, potassium phosphate/sodium phosphate (250 mg phosphorus) every 3 hours up to 8 times daily. He was given ergocalciferol 50,000 IU once weekly for his vitamin D deficiency which improved his serum 25(OH)D to 27 ng/mL. He is maintained on calcitriol 0.75 micrograms twice daily and phosphorous 225 micrograms 4 times per day. His pain is improved and takes only occasional tramadol for musculoskeletal pain. He has since been doing well, suffering only from occasional musculoskeletal pain, not interfering with his daily activities.

## 3. Discussion

### 3.1. Osteopetrosis

Osteopetrosis comprises a clinically and genetically heterogeneous group of conditions that share the hallmark of increased bone density on radiographs. Increased bone density may be a normal finding when present throughout the skeleton in gymnasts and other athletes. However, asymmetric increase in bone density or elevation much higher than normal is usually associated with a certain underlying pathology. The increased bone density in osteopetrosis results from abnormalities in osteoclast differentiation or function [1].

There are several distinct types of osteopetrosis, of which the 2 most common are the benign autosomal dominant type and the recessive malignant type [1]. Osteopetrosis produces heterogeneity of symptoms, depending on the specific mutation underlying each form of the disease [1].

The autosomal recessive (ARO) type usually manifests in infancy, causing anemia, leucopenia, hepatomegaly, and failure to thrive as well as cranial nerve symptoms, sometimes leading to early death [2]. A variant of ARO occurs secondary to a defect in carbonic anhydrase II enzyme (CAII), known as the “marble brain syndrome” [3]. It has a milder course, with concomitant renal tubular acidosis (RTA) and cerebral calcifications [3]. Other clinical manifestations comprise an increased frequency of fractures, short stature, dental abnormalities, cranial nerve compression, and developmental delay [3]. Neural failure in osteopetrosis, manifested as mental retardation and visual and hearing loss, is most likely secondary to nerve compression by narrowing of the nerve foramina [4]. ARO patients usually had a high bone alkaline phosphatase (BALP) [2]. Tubular acidosis has also been described secondary to a double gene mutation, one in the ATP6i gene, and the other in the ATPV1B1 gene encoding for the kidney specific B1 subunit of the vacuolar H^+^-ATPase [5]. Laboratory evaluation in our patient did not reveal any evidence of RTA. He did not suffer from mental retardation and his skeletal survey did not reveal any fractures.

The autosomal dominant type, also known as Albers-Schonberg disease, has 2 major subtypes, namely, ADO-I and ADO-II, ADO II being the more common form. It has been suggested that mutations in low-density lipoprotein receptor-related protein 5 (LRP5), genes important for osteoclast function, are possibly associated with ADO-I [6, 7] whereas mutations in the CLCN7 gene were recently noted to underlie ADO-II, producing a wide spectrum of disease severity [8, 9].

 These 2 forms are distinguished by radiographic and clinical characteristics. ADO-II is characterized by end-plate thickening of the vertebrae and endobones in the pelvis, whereas in ADO-I patients typically have pronounced osteosclerosis of the cranial vault. ADO-I are not likely to experience fractures [6, 10]. It has also been noted that ADO-II patients have an elevated creatinine kinase BB [11], an elevated serum tartrate phosphatase, and a low BALP. Our patient did not exhibit classic radiographic features of ADO-I or ADO-II. Our patient did have a more benign presentation at a later age. This is suggestive of a milder form of ADO.

### 3.2. Hypophosphatemia

The patient suffers from hypophosphatemia, a condition that can be caused by decreased intestinal absorption, internal redistribution, or increased urinary excretion (Table 1). Our patient demonstrated increased urinary losses of phosphorus which focused our attention to evaluate phosphaturia. Phosphate wasting in the kidneys can be secondary to an increased level of phosphaturic agents such as parathyroid hormone or diuretic use, namely, loop and thiazide diuretics. It can also be secondary to an intrinsic deficiency in phosphate reabsorption at the levels of phosphate channels in the kidneys. Two major circulating proteins are involved in the regulation of phosphorus handling by the kidney. Fibroblast growth factor-23 (FGF 23) is derived mainly from bone stromal cells and functions as a protein that increases renal phosphate excretion by reducing expression of the renal sodium-phosphate cotransporters NaPi-IIa and NaPi-Iic. FGF23 also inhibits the 1-alpha-hydroxylase, the enzyme responsible for converting 25(OH)D to 1,25(OH)_2_D, resulting in decreased 1,25(OH)_2_D production and decreased intestinal absorption of calcium and phosphorus [13]. FGF 23 is usually cleaved by the PHEX protein, resulting in its inactivation. Mutation of the FGF23 protein which causes FGF23 to be resistant to cleavage by PHEX, occurring in autosomal dominant hereditary rickets, can result in increased circulating FGF 23 and a similar biochemical profile as oncogenic osteomalacia. Mutations in the PHEX gene occurring in X-linked hypophosphatemic rickets can cause a PHEX gene product to be unable to cleave FGF 23 leading to increased FGF 23, hypophosphatemia, and hyperphosphaturia. Mutations in the kidney sodium phosphate channel as occurring in hereditary hypophosphatemic rickets with hypercalciuria can result in phosphate wasting independent of FGF23 [14].

## 4. Coexisting Increased Bone Density, Hypophosphatemia, and Phosphaturia

Our patient presented with the paradox of increased bone density with decreased bone strength, manifested as an elevated BMD with a T11 vertebral fracture. His films did not reveal any of the classic radiographic signs to differentiate between ADO-I or ADO-II. The patient also had hypophosphatemia and urine phosphate wasting. Laboratory evaluation for a potential PHEX gene mutation was negative. The patient's PTH was within normal limits and he did not use any diuretics, making these less likely on our differential. He also suffered from vitamin D deficiency, but did not have any clinical evidence of rickets.

A possible working diagnosis was osteopetrorickets, a disorder has only been described in a handful of case studies, mostly in newborn children with ARO. Five cases reported by Kaplan et al. presented with increased bone density in infancy, more than 5 SD above normal bone density, further increasing to more than 7 SD above normal when rickets was treated with calcium and vitamin D supplementation. All patients were hypophosphatemic and hypocalcemic and presented with elevated levels of alkaline phosphatase, acid phosphatase, c-terminal PTH, and 1,25-dihydroxyvitamin D. Urinary calcium/creatinine ratio was also markedly depressed and serum calcium × phosphorous product was below 30 in all children at the time of diagnosis, rising to above 40 by the time rickets had resolved. Four patients had improvement in their symptoms (less lethargy, increased mobility and activity, and stimulation of appetite with treatment with calcitriol, vitamin D, and calcium whereas the fifth patient was cured with a bone marrow transplantation [15]. Nutritional rickets caused by lack of calcium or vitamin D is not usually associated with osteopetrosis due to the poor mineralization of bone. The association between rickets and osteopetrosis has been reported in populations where consanguinity is prevalent [16]. However, rickets can also be a paradoxical complication of infantile osteopetrosis, resulting from the inability of the osteoclasts to maintain a normal calcium-phosphorus balance in the extracellular fluid. Despite markedly positive total body calcium, the serum calcium and phosphorus may not be sufficient to mineralize the newly formed osteoid leading to rickets [16–18]. The patient in this case does have a low calcium × phosphorous product (21.75), and his 24-hour urine showed phosphate wasting thus it is unlikely the patient has osteopetrorickets.

Another potential diagnosis in our patient includes ARO secondary to CAII deficiency. However, he does not manifest any evidence of renal tubular acidosis with normal serum bicarbonate ranging from 28 to 31 mmol/l. There have been reported cases of CAII deficiency without RTA, but that class of patients suffered from mental retardation. Our patient has normal mental development; he graduated from college and is a social worker. Moreover, he has no bone fractures or severe dental malocclusion [19, 20].

## 5. Conclusion

We presented a case of a young man presenting with both osteopetrosis and hypophosphatemia. He did have vitamin D deficiency but never suffered clinically from rickets. He also did not have radiologic findings suggestive of osteomalacia. The biochemical evaluations of this patient was remarkable for mild anemia, increased alkaline phosphatase levels, low plasma phosphate level with phosphate wasting in urine, and radiologically increased bone mineral density. The patient did not have a PHEX gene mutation. The patient presented at a later age, and responded well to therapy. He also lacked the classical findings for autosomal recessive osteopetrosis, which is more severe and presents at a much earlier age. Given these findings, it is likely that our patient suffers from a mild form of autosomal dominant osteopetrosis. Unfortunately, we did not have DNA testing available to confirm our clinical suspicion. Our patient has reported improved skeletal pain with calcium and phosphorous supplementation.

## Figures and Tables

**Table 1:** Hypophosphatemia [12].

Decreased intestinal absorption	Internal Redistribution	Increased urinary excretion
Secretory diarrhea	Respiratory alkalosis	Hyperparathyroidism
Steatorrhea	Refeeding of malnourished patients	Fanconi syndrome
Vitamin D deficiency/resistance	Diabetic Ketoacidosis	X-linked hypophosphatemia
Phosphate binders	Rapid cell proliferation/uptake	Autosomal dominant hypophosphatemic rickets
Severe dietary phosphate restriction	Hungry bone syndrome	Tumor associated osteomalacia
		Fibrous dysplasia
		Diuretics (loop, thiazide, CAI)
		Kidney transplant
		PTHRP

*Table adapted from [12].
